# Beyond Removal: Strategies for Sustainable Control of Water Hyacinth in Tropical Freshwater Ecosystems

**DOI:** 10.1007/s00267-026-02494-1

**Published:** 2026-05-20

**Authors:** Desalegn Chala, Diress Tsegaye, Habtamu Alem, Belachew Asalf, Melesse Eshetu Moges, Nega Tassie Abate, Ayalew Wondie, Aklilu Tilahun Tadesse, Abebayehu Aticho, Alemu Gonsamo, Lanhui Wang, Erick Lundgren, Jeffrey Kerby, Jens Christian Svenning

**Affiliations:** 1 Natural History Museum, University of Oslo, Oslo, Norway; 2 Center for Biodiversity Dynamics in a Changing World (BIOCHANGE), Department of Biology, Aarhus University, Aarhus, Denmark; 3 Section for Ecoinformatics and Biodiversity, Department of Biology, Aarhus University, Aarhus, Denmark; 4 Department of Landscape Monitoring, Survey and Statistics Division, Norwegian Institute of Bioeconomy Research (NIBIO), Ås, Norway; 5 Department of Economics and Society, Norwegian Institute of Bioeconomy Research, Ås, Norway; 6 Division of Biotechnology and Plant Health, Norwegian Institute of Bioeconomy Research (NIBIO), Ås, Norway; 7 Faculty of Science and Technology, Norwegian University of Life Sciences (NMBU), Ås, Norway; 8 Department of Biology, Bahir Dar University, Bahir Dar, Ethiopia; 9 Lake Tana and Other Waterbodies Protection and Development Agency, Bahir Dar, Ethiopia; 10 Norwegian Veterinary Institute, Research Aquatic Biosecurity, Bergen, Norway; 11 Department of Geography, System Dynamics Group, University of Bergen, Bergen, Norway; 12 Department of Natural Resource Management, College of Agriculture and Veterinary Medicine, Jimma University, Jimma, Ethiopia; 13 Threatened Species Conservation (TSC), Addis Ababa, Ethiopia; 14 School of Earth, Environment & Society, McMaster University, Hamilton, ON Canada; 15 Department of Physical Geography and Ecosystem Science, Lund University, Lund, Sweden; 16 Aarhus Institute for Advanced Studies, Aarhus University, Aarhus, Denmark; 17 Center for Ecological Dynamics in a Novel Biosphere (ECONOVO), Department of Biology, Aarhus University, Aarhus, Denmark

## Abstract

Water hyacinth is among the world’s most damaging aquatic invasive plants, forming dense mats that disrupt ecosystem functioning, fisheries, navigation, and livelihoods across tropical and subtropical freshwater systems. Its rapid spread is driven by clonal propagation, short life cycles, and prolific seed production, particularly under nutrient-enriched conditions. Although mechanical, chemical, and biological control methods are widely applied, their long-term effectiveness remains uncertain when underlying eutrophication persists. Here, we present a large-scale, one-time water hyacinth removal campaign in Lake Tana, Ethiopia’s largest lake and a UNESCO Biosphere Reserve, as a representative nutrient-rich tropical freshwater system. Using high-resolution satellite imagery, we quantified coverage one month before removal, one month after removal, and one year later. We integrated SWOT (Strengths, Weaknesses, Opportunities, Threats) analysis with a socio-ecological system map to assess mitigation mechanisms and identify sustainable management pathways capable of providing long-term solutions to halt water hyacinth proliferation in freshwater bodies. The campaign removed over 75% (~1271 ha) of water hyacinth, yet within one year the plant resurged to levels ~18% higher than pre-removal. This rebound highlights the ecological resilience of water hyacinth and the limitations of short term, noncontinuous control strategies. Our analysis identifies unmanaged catchment nutrient inputs as the primary driver of proliferation. Lake Tana serves as a model system demonstrating that water hyacinth functions less as a traditional invader and more as a bioindicator of eutrophication. We propose a transferable conceptual and methodological framework combining continuous removal, catchment-based nutrient management, and circular bioeconomy approaches, offering globally relevant lessons for sustainable management of nutrient-enriched tropical freshwater systems.

## Introduction

Freshwater resources are of critical importance to both natural ecosystems and human development. Despite their ecological and economic importance, freshwater ecosystems are widely exposed to degradation and transformation, mainly due to human interventions. Drivers of ecosystem change include direct drivers (e.g., climate change, invasive species, nutrient pollution, and overexploitation) and indirect drivers such as demographic, economic, sociopolitical, technological, and cultural drivers (Rahel and Olden 2008; Havel et al. 2015). A wide variety of taxonomic groups are considered among the top threats to freshwater bodies, and this problem is increasing globally (Paini et al. 2016). It is, however, exacerbated in tropical regions, particularly in tropical Africa due to trade routes and globalization making tropical freshwater systems critical case studies for understanding invasion dynamics. These challenges are expected to increase in the future as climate change and other anthropogenic factors may further facilitate the spread of alien species (Hoveka et al. 2016; Mungi et al. 2025).

Water hyacinth (*Pontederia crassipes* Mart.) is widely regarded as one of the most invasive aquatic plants in the tropics and subtropics (Karouach et al. 2022). It is considered a major driver of substantive ecological changes with socioeconomic consequences (Vilà et al. 2011; Harun et al. 2021). Water hyacinth is native to South America and was first introduced to North America in the late 19th century (Brown et al. 2020). It was further introduced to Europe and the tropical regions of Africa, Asia, and Australia, beginning in the early 20th century (Yan et al. 2017). It is listed by the International Union for Conservation of Nature as one of the world’s 100 worst invasive species (Téllez et al. 2008) and as one of the top 10 worst weeds in the world (Shanab et al. 2010; Gichuki et al. 2012; Patel 2012).

The biological traits contributing to the rapid proliferation of water hyacinth include a high vegetative reproduction rate, long seed dormancy, and prolific seed production (Penfound and Earle 1948). High developmental plasticity and rapid growth rates enable water hyacinth to outcompete other organisms. Water hyacinth biomass can increase rapidly under favorable conditions, particularly when the water temperature is between 27 °C and 33 °C (Mujere 2016). In phosphate- and nitrate-rich water bodies, plants grow rapidly and can quickly cover water surfaces (Ajithram et al. 2021). The proliferation of water hyacinth in many freshwater ecosystems, particularly in the tropics, has led to ecological, economic, public health, and agricultural problems (Gezie et al. 2018). Water hyacinth often forms dense, interlocking mats due to its rapid reproductive rate and complex root system, which shades the water column, limiting light penetration and thereby reducing phytoplankton growth, with cascading effects on organisms at higher trophic levels (Ajithram et al. 2021). Furthermore, it obstructs fishing and navigation, can physically jam and block hydropower turnbines, disrupt irrigation and drainage systems, and its decay can cause anoxic conditions, resulting in the death of many organisms (Kateregga and Sterner 2009; Abba and Sabarinath 2025). Water surfaces covered by water hyacinth are reported to have higher evapotranspiration than open water (Van Der Weert and Kamerling 1974; Ali and El-Din Khedr 2018). Thus, water hyacinths can potentially dry up smaller lakes and ponds and minimize water flows in rivers and irrigation canals (Van Der Weert and Kamerling 1974; Ali and El-Din Khedr 2018). Moreover, it creates a suitable habitat for breeding mosquitoes and snails, resulting in favorable conditions for the spread of vector-borne diseases such as malaria and bilharzia (Barber and Hayne 1925; Ofulla et al. 2010).

Various strategies have been employed worldwide to manage the spread of water hyacinth in freshwater ecosystems. Physical (i.e., mechanical and manual removal), chemical (herbicides), and biological (utilizing natural enemies of water hyacinth, such as *Neochetina* spp. weevils) control methods (applied in isolation or together) are the most commonly used approaches (Karouach et al. 2022). However, these interventions have proven ineffective for two primary reasons. First, a single water hyacinth plant can produce thousands of seeds, which can remain viable in sediments for ~20–30 years (Yang et al. 2025). Second, water hyacinth is capable of vegetative propagation, allowing it to easily regenerate from propagules in nutrient-rich environments and rapidly proliferate, rendering the interventions futile. These characteristics, combined with its responsiveness to nutrient-rich conditions, render water hyacinth especially capable of rapid proliferation compared to other invasive aquatic plants. Based on these considerations, we hypothesize that rapid proliferation of water hyacinth in large water bodies of warm tropical regions primarily reflects underlying water quality deterioration driven by eutrophication. This perspective suggests that, in such systems, water hyacinth responds more strongly to nutrient pollution than factors typically associated with invasive species dynamics, thereby acting as a symptom of ecosystem degradation rather than solely an invasion problem. Therefore, the most sustainable solution is to improve water quality through catchment area management, thereby minimizing the influx of nutrients. Consistently removing plants and utilizing them for various purposes can also aid in nutrient extraction through phytoremediation, thereby reducing nutrient levels in freshwater bodies (Wang et al. 2013).

Here, we analyze a one-time, but large-scale, water hyacinth removal campaign from Lake Tana in Ethiopia, which took place from November to December 2020. The lake was selected primarily because the removal event provides a unique opportunity to examine how invasive plant management unfolds in nutrient-rich tropical freshwater systems, offering insights applicable to global contexts. Our main goal is to highlight the limitations of relying solely on noncontinuous physical removal as an intervention strategy and to extract lessons relevant for other tropical lakes worldwide. To accomplish this, we utilized satellite observations during three distinct time frames: approximately one month prior to the campaign (October 2020), approximately one month after the campaign (January 2021), and one year following the campaign (December 2021). Additionally, we utilize a Strengths, Weaknesses, Opportunities, and Threats (SWOT) analysis to examine existing methods for mitigating the water hyacinth issue, propose when to use each approach, recommend a lasting solution, and test our hypothesis. These methods include physical removal, biological control, chemical intervention, and catchment area management. To further strengthen our perspective, we present a comprehensive system map that examines the interconnections and feedback loops among the factors that influence the spread of water hyacinth in freshwater environments, highlighting generalizable principles for managing nutrient-driven invasions.

## Materials and Methods

### Water Hyacinth Removal Campaign in Lake Tana

**Fig. 1:**
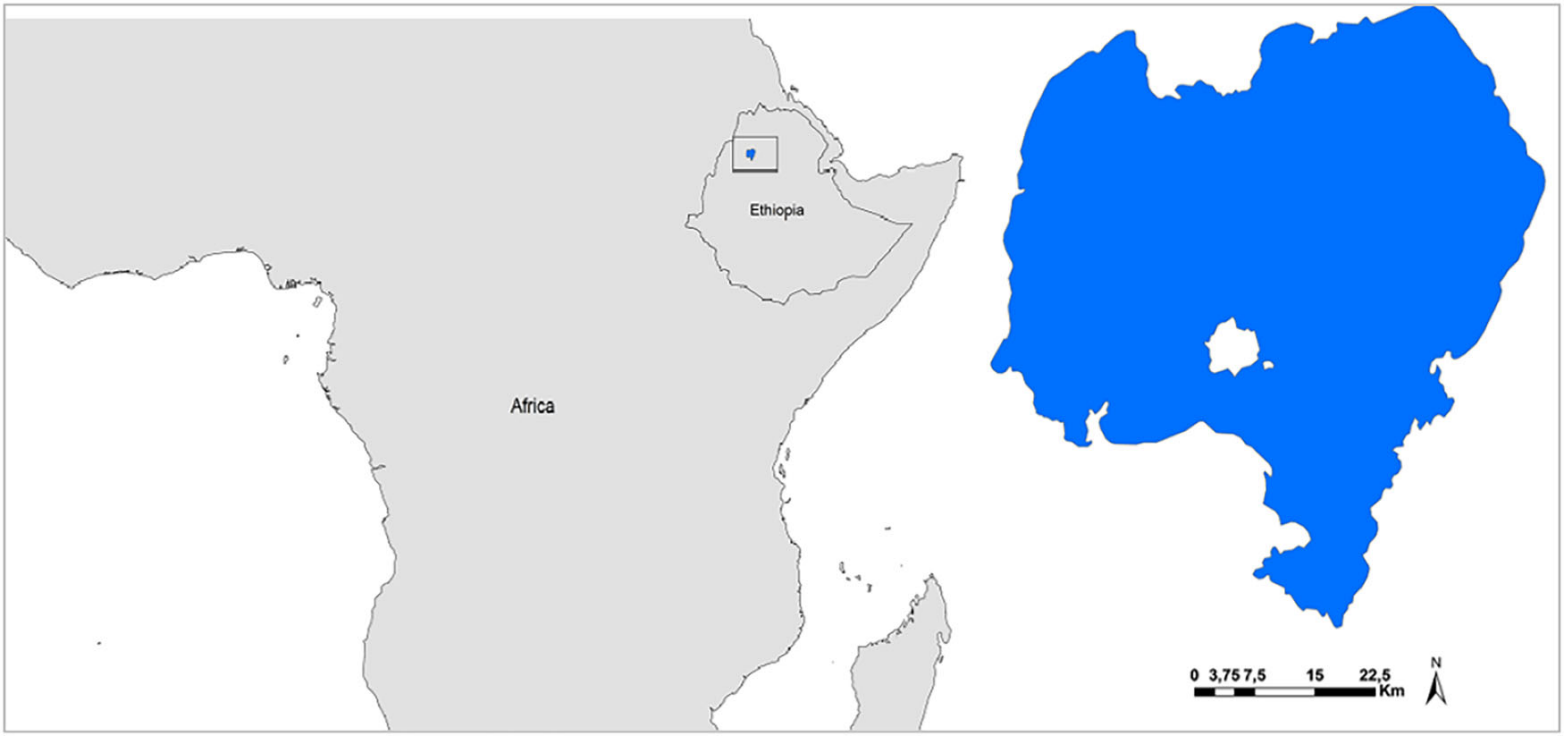
Lake Tana, Ethiopia. The spatial distribution of water hyacinth and the locations of removal campaigns are primarily in the northern and eastern parts of the lake and are shown in detail in Figs. 2–4

Lake Tana is the headwater of the Blue Nile and the largest lake in Ethiopia (Fig. 1). It has an area of over 3000 km² and a catchment area of ~15,000 km². The lake harbors substantial biodiversity, ancient churches and monasteries located on its islands and is one of the UNESCO World Heritage sites. Its invasion by water hyacinth was first reported in 2011, and it immediately began expanding over the lake’s surface, affecting the livelihoods of local people who rely on the lake for fishing and other economic benefits (Enyew et al. 2020; Abba and Sabarinath 2025).

More than 40 rivers and streams flow into Lake Tana from its large surrounding catchment area (Stave 2017). It has 28 species of fish, of which 21 are endemic flocks of Labeobarbus (Damesé 2012; Getahun and Dejen 2012; Stave 2017). The lake provides essential ecosystem services and contributes significantly to food security, livelihoods, and national economies through the direct exploitation of fisheries, water resources for irrigation, recharge of groundwater, hydropower generation, transport, recreation and tourism, microclimate regulation, and habitat for a large variety of birds and other wildlife, such as hippopotamus (Enyew et al. 2020). In recognition of the lake’s rich biodiversity, UNESCO recognized the lake as part of its World Network of Biosphere Reserves in June 2015 (Asmare et al. 2020). Water hyacinth has invaded over 190 km of the perimeter of Lake Tana (Worqlul et al. 2020). If this spread is not abated, it can halt many economic activities on water bodies and have a drastic impact on the lake ecosystem.

The invasion of Lake Tana by water hyacinth has been a prominent issue, garnering significant attention from various media outlets (Mekonen et al. 2016; Kibret 2017). Numerous initiatives have been undertaken to address this problem. Both the federal and provincial governments have made efforts to combat the spread of water hyacinth by mobilizing local communities and utilizing both manual labor and mechanical harvesters. The collective efforts of local communities led to a large-scale removal campaign in the most severely affected areas of Lake Tana, particularly in the northern and northeastern zones. Conducted over a 30-day period between November and December 2020, the intervention was reported to have cleared around 80% of the infestation. In this paper, we assess the effectiveness of that campaign using satellite-based observations.

### Satellite Observations for Classification and Change Detection

We acquired a series of Copernicus Sentinel-2 MSI L2A satellite observations of Lake Tana from the Google Earth Engine API (Gorelick et al. 2017) for October 2020 to December 2021. We selected cloud-free Sentinel-2 data acquired one month prior to the start of the water hyacinth removal campaign in October 2020, as well as roughly a month (January 2021) and one year (December 2021) after the intervention campaign. The satellite observations cover the whole lake water body, excluding areas outside the lake.

To quantify the spatial extent of water hyacinth invasion in each Sentinel-2 observation, we used a supervised classification and regression tree (CART) (Breiman et al. 1984) machine learning algorithm in Google Earth Engine (GEE, classifier.smileCART) over the entire lake surface. This was done separately for each image using training datasets of the ‘water hyacinth’ and ‘other’ classes (water, silty water, vegetation), produced by visual interpretation of the high-resolution Sentinel imagery. The training data were further evaluated against previously published water hyacinth cover maps from the same periods (Worqlul et al. 2020) and very high-resolution (3 m) Planet Labs satellite imagery. Classifications were run using four Sentinel-2 spectral bands: 2 (Blue), 3 (Green), 4 (Red), and 8 (near-infrared), on a random sample of 75% of the training data for each scene. Classification performance and accuracy were evaluated using confusion matrices generated from a random sample of 25% of the training data for each scene. To minimize the impact of misclassifying small pixel groups, classifications were generalized and restricted to the core region of the water hyacinth outbreak in the northeast corner of the lake.

The classification of satellite observations from all three periods achieved high accuracy, with an overall classification accuracy of 90.4% across all dates and classes. The water hyacinth class specifically had an overall recall (consistent accuracy) of 89.4% and an overall precision (i.e., the proportion of predicted water hyacinth pixels that were correctly classified) of 97.1% across the dates.

### Strengths, Weaknesses, Opportunities, and Threats (SWOT) Analysis

The origins of SWOT analysis can be traced back to strategic management research conducted in the 1960s and 1970s (Sevkli et al. 2012). SWOT, an acronym for Strengths, Weaknesses, Opportunities, and Threats, has become a popular tool for evaluating different strategies and determining the most suitable strategy for a given business environment (Pickton and Wright 1998; Sevkli et al. 2012). The underlying concept revolves around the idea that the performance of an economic entity, typically a business, in achieving specific goals is influenced by the interaction between its management, internal characteristics, and the external context in which it operates. While the entity may not have immediate control over external factors, these factors play a significant role in shaping its outcomes (Houben et al. 1999). SWOT analysis has found extensive application in various fields, including ecology, with a particular focus on sustainability, ecosystem services, and tourism (Weyns 1995; He 2014; Bull et al. 2016; Wang et al. 2020). Here, we adapted this approach to evaluate and compare different intervention mechanisms for mitigating the invasion of water hyacinth in freshwater bodies.

First, we identified the mitigation mechanisms employed to control water hyacinth. Then, we adopted a systematic approach to commonly used mitigation mechanisms, including, but not limited to, mechanical, chemical, biological, and catchment area management. The assessment of the Strengths, Weaknesses, Opportunities, and Threats (SWOT) for each mitigation mechanism was conducted using expert judgment by the co-authors, who have complementary expertise in ecological modeling, plant pathology, aquatic ecology, plant ecology, and invasive species management. The SWOT analysis was primarily grounded in published literature, expert knowledge, and interpretative synthesis rather than directly tested hypotheses.

Strengths were evaluated based on inherent advantages and positive performance characteristics, including effectiveness in reducing water hyacinth biomass, cost-efficiency over short- or long-term implementation, ecological sustainability, ease of deployment, scalability across spatial contexts, adaptability to varying environmental conditions, speed of action, technical complexity, and compatibility with existing management practices. Weaknesses were identified by analyzing potential limitations and challenges directly associated with each mitigation mechanism, including high costs, limited effectiveness, operational difficulties, environmental impacts (e.g., habitat disturbance, non-target effects), and constraints on implementation. Opportunities were assessed by reviewing literature and expert knowledge to identify factors that could enhance the effectiveness, efficiency, or scalability of mitigation mechanisms. This included evaluating emerging technologies, innovative approaches, supportive policy frameworks, and recent research advancements, allowing us enhance the effectiveness and efficiency of mitigation mechanisms. Threats were analyzed to account for potential risks arising from external or uncertain factors, including unintended environmental impacts, regulatory constraints, social acceptability, economic feasibility, and the potential re-emergence or spread of invasive species. All assessments were based on a combination of literature review and structured expert judgment. Co-authors cross-validated their assessments to reduce bias and ensure a balanced evaluation (Sevkli et al. 2012). This approach allowed us to systematically synthesize existing knowledge and place the Lake Tana case study within a broader management context.

## Results

### Impact of Physical Removal on Water Hyacinth Coverage

**Fig. 2:**
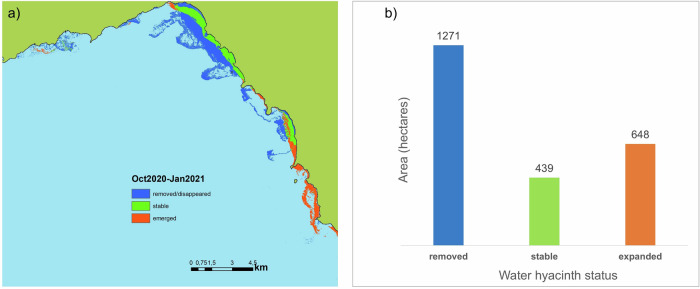
Overlay of classification results from satellite observations acquired about one month before and after the removal campaign in October 2020 and January 2021, respectively (**a**). The total area (in ha) from which the plants were removed, remained (stable), and the new areas to which the plants expanded after a month of removal are presented in (**b**). Oct – October, Jan – January. The black line shows the lake boundary. See also Fig. 4

Our analysis of satellite observations, using images acquired approximately one month before and one month after the intervention showed that the campaign in November/December 2020 removed more than 75% of the water hyacinth cover, spanning over 1271 ha (Fig. 2).

**Fig. 3:**
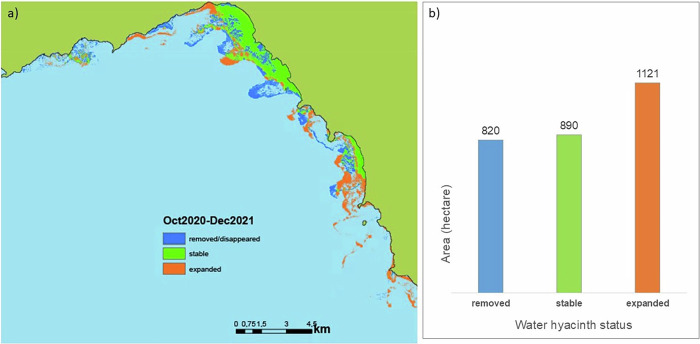
Overlay of classification results from satellite observations acquired approximately one month before and a year after the removal campaign in October 2020 and December 2021, respectively (**a**). The area (in ha) from which the plants were removed (present in October 2020 but not present in December 2021), stable (present during both periods) and areas to which the plants expanded after a year are presented in (**b**). Oct- October, Dec – December. The black line shows the lake boundary

**Fig. 4:**
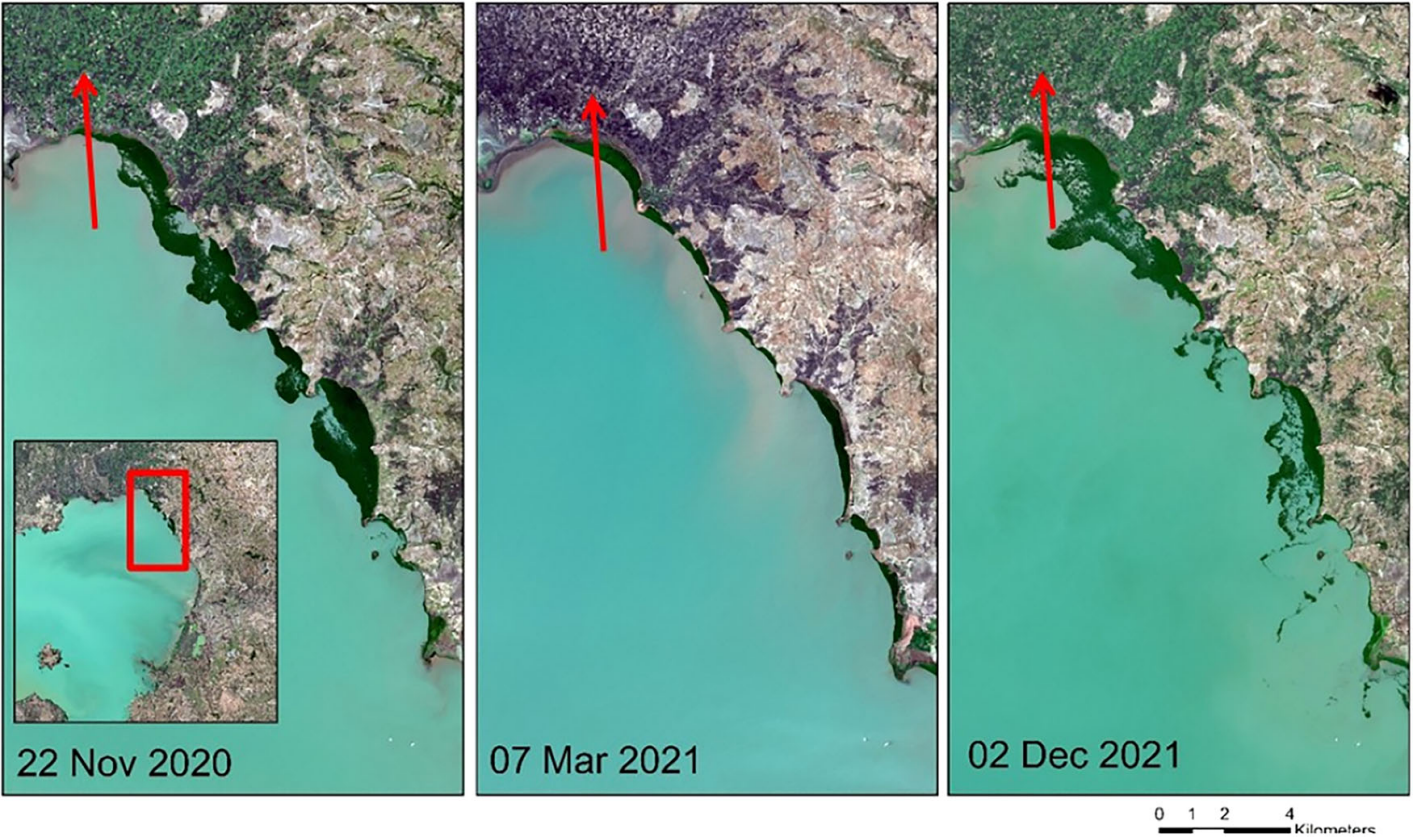
Example of satellite data from Sentinel-2 acquired three days after the intervention started (22 November 2022), approximately two months after the campaign ended (07 March 2021) and approximately one year after the campaign (02 December 2021) in Lake Tana, Ethiopia. The disposal ground for water hyacinth is visible in the image (indicated by the dark green shade along the northwestern boundary of the lake, indicated in red arrow

Our findings showed that the area covered by water hyacinth one month before the time of intervention was 1710 ha but had expanded to 2011 ha one year after the intervention, i.e., an ~18% increase (Fig. 3). Although water hyacinth reclaimed areas occupied prior to the intervention, the mechanical removal process produced fragments (propagules) that could drift with wind and water currents (Fig. 4). These propagules likely contributed to the colonization of areas where water hyacinth had not been present prior to the intervention campaign (Figs. 2 and 3).

### SWOT Analysis

Physical methods, such as manual and mechanical removal, do not necessarily require technical expertise, especially when manual removal is employed. Water hyacinth is known for its capacity to uptake nutrients from the water, and its removal can contribute to nutrient reduction through biomass extraction, a process often associated with phytoremediation (Wang et al. 2013). The approach helps to enhance ecological health by opening water surfaces and providing improved breeding grounds for fish and various other life forms (Kateregga and Sterner 2009; Abba and Sabarinath 2025; Mujere 2016). Additionally, the extracted biomass has the potential to be utilized for bioenergy production (such as biogas), compost production, paper manufacturing, basket weaving, furniture production, and other similar endeavors (Abba and Sabarinath 2025; Yang et al. 2025). However, the large-scale integration of this biomass into industrial processes remains limited and depends on the availability of appropriate infrastructure, market demand, and institutional coordination. Therefore, these uses should be considered as potential opportunities rather than established outcomes. Utilizing the removed biomass of water hyacinths as raw material inputs for industrial, agricultural, environmental, and household applications could promote a sustainable cycle of water hyacinth removal by reducing running costs and boosting the local economy (Mujere 2016).

**Fig. 5:**
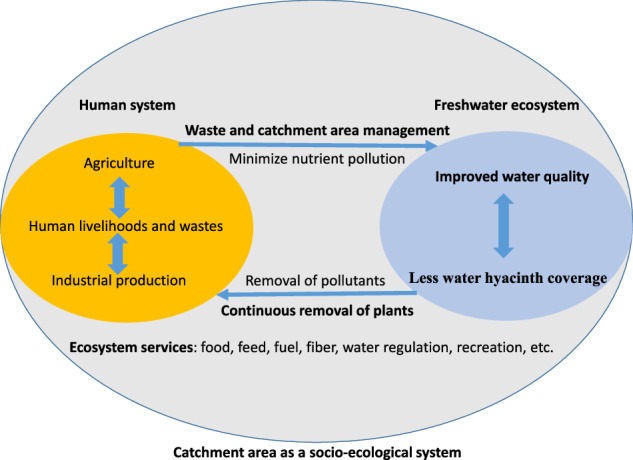
An integrated and socio-ecologically sustainable solution to global water hyacinth invasions of freshwater ecosystems

However, manual and mechanical removal methods can be labor-intensive, time-consuming, and expensive, particularly when dealing with extensive hyacinth populations. They may not eliminate all plant materials, which can lead to regrowth. Furthermore, dense water hyacinth mats are known to serve as breeding grounds for mosquitoes and snails that act as vectors for parasites causing malaria and schistosomiasis (also known as bilharzia), which may pose public health risks during infestation and management activities (Ofulla et al. 2010). Without continuous implementation, these methods only yield short-term benefits (Table 1, Fig. 5).

**Table 1:** Strengths, Weaknesses, Opportunities, and Threats (SWOT) analysis of different mitigation mechanisms of water hyacinth

1. Physical control (manual and mechanical)	
Strengths	Weaknesses
• Does not require technical experts and limited adverse environmental impacts, specially when manually removed • Improves water quality by removing excess nutrients along with plant biomass (phytoremediation). • Can also remove toxins from the water. • Opens up the water surface, creating suitable conditions for reproduction of fish and other aquatic life. • Facilitates fishing, transportation, and other economic activities.	**Manual harvesting** • Labour- and time-intensive; inefficient to uproot and transport. • Finding a disposal site for biomass is challenging. • Leftover stolons are likely to remain and can regrow. **Mechanical harvesting** • High investment and running costs. • Causes ecosystem disturbances and noise. • Inefficient in shallow areas along shores where water hyacinth commonly grows. • Leftover stolons are likely to remain and can regrow. **Improper disposal** • Discarded biomass can serve as a recruitment source, potentially promoting reinvasion or proliferation of other invasive species.
**Opportunities**	**Threats**
• Compost, paper, biogas, and household furniture can be produced from harvested biomass. • Enhances participation of local communities. • Contributes to job creation.	• Water hyacinth serves as a breeding ground for mosquitoes and snails. People involved in removal may face health hazards, including exposure to vector-borne diseases such as malaria and bilharzia.
**Overall assessment**: It can be efficient in the short term if the removal occurs continuously. If coupled with the catchment area management, it can ultimately produce a sustainable solution (see below).	
2. **Biological control**	
**Strengths**	**Weaknesses**
• Potentially environmentally friendly. • Low operational costs once insects are released. • Can be self-sustaining if insect populations establish successfully. • Certain weevils (e.g., *Neochetina eichhorniae*) are highly specific to water hyacinth and do not affect other organisms.	• High initial investment cost for introducing insects. • Requires specialized skills and knowledge. • Dead biomass sinks to the bottom, creating anoxic conditions that can harm other organisms and the ecosystem. • May increase nutrient availability, potentially promoting further water hyacinth blooms. • Inefficient if habitat is unsuitable and insects do not reproduce rapidly.
**Opportunities**	**Threats/Risks**
• It provides the possibility to select locally available biological enemies, including fungi and insects.	• Regulatory processes to approve, import, and release insects can be slow.
**Overall assessment**: Can be used as an alternative when the rate of physical removal and water hyacinth expansion rate do not match, but cannot produce a lasting solution.	
3. **Chemical control**	
**Strengths**	**Weaknesses**
• Can cover large areas efficiently. • Effective in a short period. • Relatively low cost.	• Not environmentally friendly. • Dead biomass sinks to the bottom, potentially creating methane and fertile conditions for water hyacinth regrowth. • Requires skilled workforce for herbicide selection, calibration, dose calculation, safe handling, transport, and disposal. • Needs approval for import and use. • Protective equipment is necessary for safe application.
**Opportunities**	**Threats/risks**
• Potential use of local knowledge, such as herbal extracts, as alternative herbicides.	• Development of herbicide-resistant water hyacinth genotypes. • Potential negative impacts on water quality and aquatic life. • Possible effects on non-target plants, other organisms, and human health.
**Overall assessment:** This can be used as an alternative when the rate of physical removal and the water hyacinth expansion rate do not match, but it is not recommended as a lasting solution.	
4. **Catchment area and waste management**	
**Strengths**	**Weaknesses**
• Minimizes pollution influx and improves water quality, creating conditions unsuitable for water hyacinth growth for a long-term solution. • Fosters a healthy and productive ecosystem within the catchment area. • Helps reduce soil erosion, increase water infiltration, and maintain wetland ecosystems. • Enhances biodiversity conservation, carbon sequestration, crop yield, and overall ecosystem health.	• Implementation is costly and time-consuming. • Involves multiple stakeholders; coordination and follow-up are challenging. • Requires knowledge, commitment, and strict follow-up.
**Opportunities**	**Threats/Risks**
Benefits the entire catchment area, including soil erosion reduction, biodiversity conservation, and improved agricultural productivity. • Potential economic opportunities if multipurpose tree plantations are integrated.	• May affect investment around the lake if investors are unwilling to share the costs of waste management. • Land-use policies may not be acceptable to local communities. • Potential conflicts with local communities, including cultural and knowledge gaps.
**Overall assessment**: An excellent option to bring sustainable solutions in the long term. It will be highly effective if coupled with continuous physical removal.	

Biological control offers an environmentally friendly approach, as certain weevil species such as *Neochetina eichhorniae* target only water hyacinth for consumption (Kariuki and Minteer 2021; Karouach et al. 2022; Mukarugwiro et al. 2023). While it requires a significant initial investment, its operational costs remain low. However, a potential drawback arises from the accumulation of dead water hyacinth and weevils’ biomass, which can sink to the lake’s depths and generate anoxic conditions detrimental to other organisms and the overall ecosystem (Mukarugwiro et al. 2023). Additionally, this process inadvertently nourishes water hyacinth, thereby enhancing its resilience by providing a natural source of fertilizer.

Herbicides, as a chemical control method, offer numerous benefits. They can efficiently cover large areas in a short time and are relatively affordable. Herbicides enable quick and precise control in specific regions. However, improper use or excessive reliance on herbicides can lead to the development of resistance in water hyacinth populations, posing challenges for future control efforts. Furthermore, the decomposed biomass of water hyacinth has the potential to accumulate and negatively affect other organisms by creating oxygen-deprived conditions (Mukarugwiro et al. 2023). Additionally, water hyacinths can easily regenerate from propagules, utilizing the decomposed plant material as a source of nutrients. Although most aquatic herbicides are designed to be plant specific, their application may involve short term restrictions on water use and can have implications for fisheries, recreation, and local livelihoods if not properly managed. In particular, when the spread rate of water hyacinth exceeds that of physical removal or biological control methods, temporarily opening water surfaces can be advantageous, promoting ecological health, fishing, and navigation.

By implementing catchment area management effectively, the root causes of water hyacinth infestation can be addressed, reducing nutrient influx and improving water chemistry. This will starve the water hyacinth and prevent its long-term proliferation. However, achieving these goals requires public awareness campaigns and educational initiatives to promote sustainable practices within catchment areas. Challenges such as changing land-use practices, urbanization, and inadequate regulatory enforcement can also contribute to ongoing nutrient pollution and the continued growth of water hyacinths. To effectively combat these issues, it is crucial to implement comprehensive and well-executed watershed management measures. By doing so, the influx of nutrients can be minimized, and sustainable control of water hyacinth can be achieved, especially when combined with consistent physical removal efforts.

Generally, the SWOT analysis is grounded in published literature, expert knowledge, and interpretative synthesis rather than directly tested hypotheses. It also accounts for the biological and ecological unlikelihood of achieving a proliferation rate comparable to that observed in Lake Tana without eutrophication. Several of the identified points would benefit from further experimental validation to strengthen their empirical basis.

**Fig. 6:**
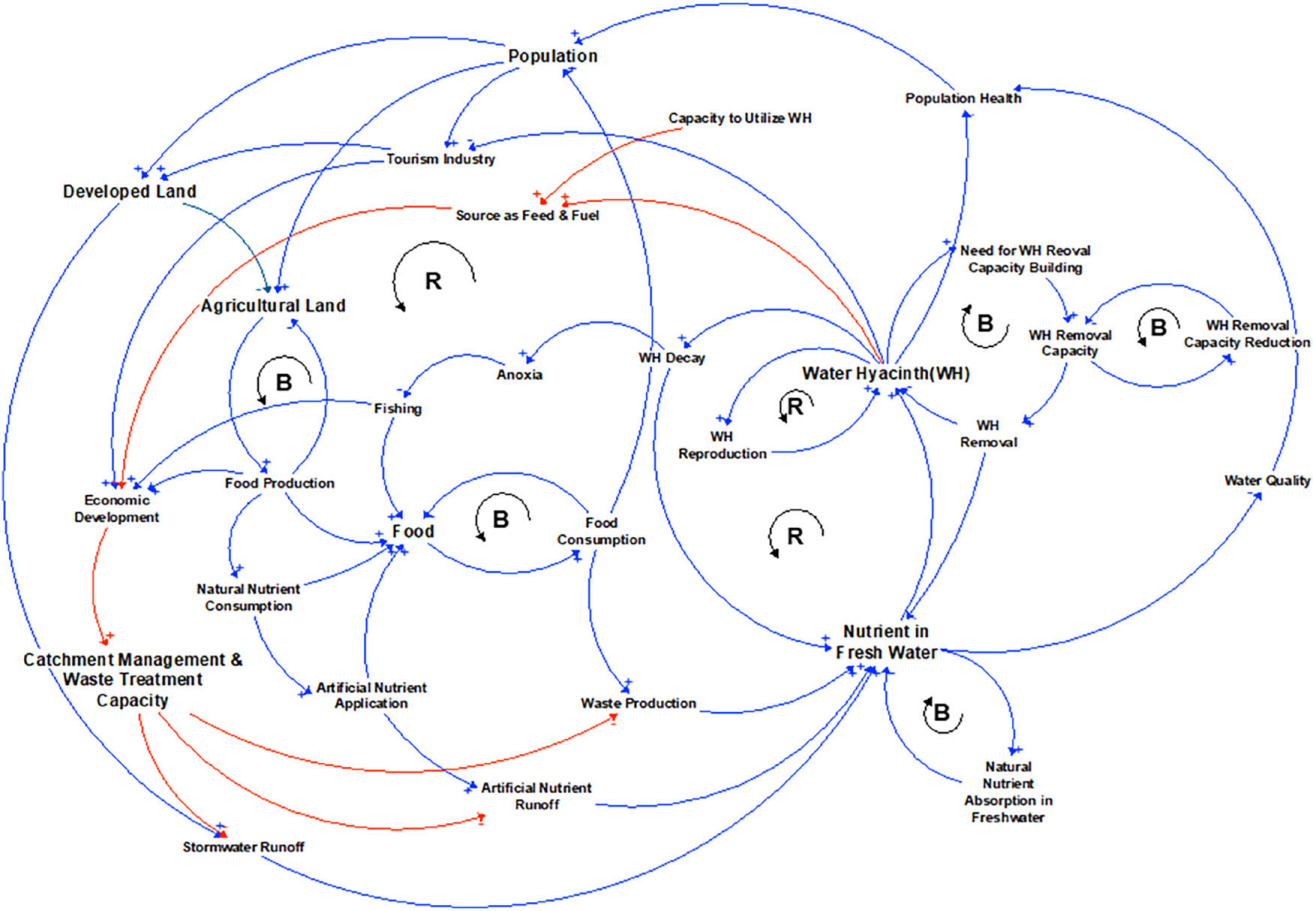
A system map for causes of water hyacinth proliferation in freshwater and potential policy for its reduction. WH stands for water hyacinth. Selected feedback loops are labeled, with “R” indicating a reinforcing feedback loop and “B” indicating balancing (counteracting) feedback. The ‘+’ and ‘−’ signs at the end of the arrows indicate the direction of increase or decrease in effect, respectively, given that everything else is kept constant. Bolded variable names indicate key factors in a causal loop diagram, where one can start to study the causal map. The red colored links indicate potential policy options that can be considered (for details please see Supplementary S1)

Our system map illustrates the relationship between the intensification of farming practices and land development, which aims to meet the needs of a growing population within the catchment area, as well as their impact on nutrient load and the proliferation of water hyacinth (Fig. 6). Additionally, the map demonstrates how the continuous removal of water hyacinth from water bodies contributes to a circular economy and facilitates nutrient removal through phytoremediation. Furthermore, it highlights the effectiveness of integrating water hyacinth removal with catchment area management to improve water quality and permanently halt the proliferation of water hyacinth.

## Discussion

Our analysis of satellite images clearly indicates that the water hyacinth removal campaign in Lake Tana successfully eliminated over 75% of the water hyacinth cover, amounting to ~1271 hectares. However, despite the initial success, the water hyacinth began to multiply immediately after the intervention, rapidly regaining control over the previously cleared areas. Within a year, the affected area expanded to 2011 hectares, representing an ~18% increase in size. While the rapid regrowth highlights the resilience of water hyacinth populations and indicates eutrophication (Musil and Breen 1977; Ripley et al. 2006; Wang et al. 2013; Ajithram et al. 2021), we lack data to quantify how different levels of eutrophication contribute to this expansion. The observed proliferation may also stem from limitations of the physical removal method, including increased water clarity and the generation of propagules that promote recolonization. Future studies should examine water hyacinth proliferation across freshwater systems with varying nutrient levels to better understand the role of eutrophication. Although the water hyacinth reclaimed its previously occupied territory in Lake Tana, the biomass appeared less dense after the intervention (see Fig. 4). This reduced density could potentially facilitate plant dispersal by wind. Consequently, it may have contributed to the spread of water hyacinth into previously unaffected areas, leading to a widespread invasion of the lake by water hyacinth. Our analysis suggests that its impact was short-term despite the considerable efforts and financial resources invested in the extensive water hyacinth removal campaign. This finding emphasizes the ecological and economic ineffectiveness of such campaigns.

The long-term effectiveness of physically removing water hyacinth is limited for several reasons. First, when the plants are removed, any leftover buds and stolons can generate new plants through asexual reproduction (Gaikwad and Gavande 2017). This means that even if the visible plants are removed, new plants can quickly emerge from the remaining plant parts (Xu et al. 2025). Second, water hyacinth is known to produce a large number of seeds, with each plant capable of producing up to 1000 seeds (Barrett 1980; Yang et al. 2025). This high seed production enables the surviving plants to rapidly produce a significant number of offspring, further contributing to the spread of water hyacinth.

Additionally, the seeds can survive in water and sediments for several years, allowing them to germinate and produce new plants whenever conditions are favorable (Penfound and Earle 1948; Cacho et al. 2006; Albano Pérez et al. 2011; Yang et al. 2025). The resilience of these seeds contributes to the persistent presence of water hyacinths in affected areas. Furthermore, during the removal process, plant biomass and seeds can easily disperse because the plants are fragile and can readily propagate asexually, making it challenging to eradicate water hyacinth. The resilience of water hyacinths is particularly pronounced in nutrient-rich freshwaters found in tropical regions, such as Lake Tana (Gezie et al. 2018), where warm temperatures and high nutrient availability create favorable conditions for water hyacinths to thrive and reproduce year-round (Yang et al. 2025).

Although biological and chemical control methods can have positive impacts in opening up water bodies and promoting ecosystem health and economic activities, it is important to acknowledge that both approaches contribute to the accumulation of dead biomass in the water. This accumulation can lead to anoxic conditions, which are harmful to biodiversity, and and may act as a nutrient source that promotes further proliferation of water hyacinth. For example, studies from Lake Victoria (East Africa) and southern India have documented rapid regrowth of invasive aquatic plants following mechanical or chemical removal when nutrient inputs were not managed (Karouach et al. 2022). Thus, it is crucial to recognize that neither biological nor chemical control alone provides a long-term solution (Karouach et al. 2022; Djihouessi et al. 2023). However, the chemical control has the advantage of covering large areas quickly, which can be useful in situations where the expansion rate exceeds the capacity for physical removal. Similarly, biological control methods can provide sustained suppression when suitable agents establish successfully, but their effectiveness depends on environmental conditions and time for population establishment (Cinco-Izquierdo et al. 2026). These case studies underscore the importance of integrating removal methods with catchment-scale nutrient management to achieve sustainable control of water hyacinth (Yang et al. 2025; Cinco-Izquierdo et al. 2026).

Considering the magnitude of these challenges, addressing the issue of water hyacinth requires an integrated approach that incorporates short, medium, and long-term solutions, with lessons applicable to tropical lakes worldwide (Djihouessi et al. 2023; Cinco-Izquierdo et al. 2026). Such an approach should be implemented within both the affected lakes and their surrounding catchment areas to tackle the problem efficiently and sustainably (Sitotaw et al. 2022; Engdaw et al. 2024). It is crucial to conduct experimentation and research to develop appropriate control and prevention measures tailored to the specific sociocultural, economic, and environmental conditions of each freshwater body affected by water hyacinth, while extracting insights that inform broader invasive species management strategies.

To complement the ongoing physical removal of water hyacinths from freshwater bodies with high biodiversity, it is recommended that measures be implemented in the catchment areas and waste management practices be improved. The former approach facilitates the removal of excess nutrients through phytoremediation, while the latter helps minimize nutrient influx, ultimately improving water chemistry and the ecological conditions that contribute to water hyacinth proliferation. Moreover, removing water hyacinth biomass should be institutionalized and integrated into a supply chain that offers economic benefits. Therefore, we recommend combining continuous removal and utilization with catchment area management as lasting solutions.

### Continuous Removal and Utilization

Continuous removal of water hyacinth offers numerous advantages. First, it opens up the water surface, enhancing ecosystem functions (Gezie et al. 2018) and promoting primary productivity (Vilà et al. 2011). Additionally, it creates reproduction grounds for fish, leading to improved fishing opportunities and navigation over water bodies (Harun et al. 2021). Second, water hyacinth serves as a phytoremediation agent, aiding in the removal of environmental pollutants (Rodríguez-Gallego et al. 2004; Villamagna and Murphy 2010; Panneerselvam and Priya K 2021). This approach is particularly effective when combined with catchment area management strategies, as illustrated in Fig. 5 and detailed in Table 1. Consequently, the overall water quality is significantly enhanced. However, continuous removal requires substantial effort, resources, and careful logistical planning, which can limit its feasibility in practice. Although the removal of water hyacinth mats increases sunlight penetration and can enhance phytoplankton production, it may also lead to unintended consequences, such as colonization by other invasive algae or harmful phytoplankton under nutrient-rich conditions. Dense water hyacinth mats are known to reduce light availability and alter ecosystem dynamics, which can influence post-removal community composition (Herrera Ollachica et al. 2025).

Lastly, water hyacinth can be utilized as a potential source of income for local communities. The biomass of water hyacinth can be converted into various eco-friendly products, such as biogas (a climate-friendly fuel), compost/green manure, animal feed supplements (using wilted water hyacinth), fish meal for aqua farming (via vermiculture), fuelwood (by burning the biomass to produce biochar), and a wide range of household handcrafts (Harun et al. 2021; Dhinesh et al. 2024).

### Catchment Area and Waste Management

The Lake Tana catchment is experiencing rapid population growth, urbanization, and land use/cover (LU/LC) changes that are significantly impacting water quality, with settlement and cropland expansion driving nutrient pollution (notably nitrate and phosphate) and microbial contamination, while forested areas help maintain healthier water conditions (Engdaw et al. 2024). Seasonal fluctuations further influence water quality, with higher concentrations of fecal and total coliforms, nutrients, turbidity, and biochemical oxygen demand observed during the wet season due to increased runoff from surrounding lands, whereas the dry season generally shows lower pollutant loads (Sitotaw et al. 2022). This strong link between LU/LC changes, seasonal dynamics, and ecosystem health underscores the urgent need for sustainable land and water management to safeguard water resources and ecosystem services (Sitotaw et al. 2022; Engdaw et al. 2024).

The rapid proliferation of water hyacinth in large freshwater ecosystems is largely dependent on, or at least strongly facilitated by, eutrophication, indicating that effectively managing the influx of nutrients into these water bodies through catchment area management is crucial for a sustainable long-term solution (Musil and Breen 1977; Engdaw et al. 2024). This management approach involves implementing various practices, such as appropriate tillage, terraces, stone/soil bunds, filter strips, grassed waterways, and agro-forestry (Bishaw et al. 2022; Keshyagol et al. 2026). In addition to watershed management through agriculture, it is also crucial to implement proper regulations and enforcement to prevent the discharge of untreated sewage and other pollutant effluents from factories and towns (Sitotaw et al. 2022). To achieve this, a comprehensive understanding of the interactions between the lake and its surrounding watershed is necessary, including identifying key pollutants and implementing suitable management strategies to address these issues. Adopting a multidisciplinary socio-ecological systems approach is crucial for addressing the water hyacinth issue and developing effective mitigation solutions. This approach evaluates and comprehends the relevant socioeconomic and environmental activities within the lake catchment, identifying those that may hinder the ongoing measures or, conversely, aid in addressing the water hyacinth problem (Engdaw et al. 2024).

To conclude, water hyacinth relies on its surrounding environment for nutrients. Its rapid growth in large bodies of water is a clear indication of eutrophication, which is driven by an influx of nutrients. Given the infeasibility of completely eradicating and permanently ceasing propagule input, improving water quality emerges as the only sustainable solution. This can be achieved by co-managing the continuous removal of water hyacinth to facilitate nutrient removal through phytoremediation, alongside catchment and waste management practices, to reduce the nutrient load. Beyond promoting ecological health, the economic benefits of sustained biomass removal include improvements in fishing and navigation, as well as the provision of reliable raw materials for industrial, agricultural, environmental, and household applications. Combining the two approaches can reduce the ecological contexts that promote water hyacinth dominance, thereby providing a critical means to manage important freshwater ecosystems sustainably.

In general, our argument centers on the following key points. First, the conventional approach of controlling the proliferation of water hyacinths in freshwater environments often fails because it overlooks a crucial ecological factor: eutrophication, which drives the abundance of these plants. With this understanding, we argue that enhancing water quality is the sole sustainable solution. This principle may also extend to other non-nitrogen-fixing aquatic weeds. Second, eutrophication leads to ecosystem deterioration, having significant impacts on biodiversity and local livelihoods. Thus, the proliferation of water hyacinths not only presents a problem but also serves as a symptom and indicator of ecosystem degradation. Third, water hyacinths possess potential benefits as a valuable resource (Nandiyanto et al. 2024). They can be utilized for composting, biogas production, furniture manufacturing, and even as animal feed (Nandiyanto et al. 2024; Yang et al. 2025). This versatility underscores that water hyacinths can be perceived not only as a problem but also as a resource. Fourth, by consistently removing them and utilizing them as a resource, we can extract nutrients from freshwater, further emphasizing their potential in addressing ecological issues. This highlights the fact that water hyacinths can also be seen as part of the solution.

This study offers several novel contributions with broader relevance beyond Lake Tana. First, it shows that water hyacinth rapid proliferation mainly serve as an indicator of nutrient enrichment in tropical freshwater systems, rather than solely treated as an invasive nuisance. Second, it introduces an integrated analytical framework combining high-resolution satellite monitoring, SWOT analysis, and socioecological system mapping to evaluate management interventions. While exploratory, this framework provides a basis for comparable assessments in other freshwater systems. Third, the study highlights the importance of coupling biomass removal with catchment-based nutrient management and resource utilization strategies, contributing to ongoing discussions on sustainable and circular approaches to invasive species management.

For future research, we recommend systematically comparing the rate of water hyacinth proliferation across invaded sites that share similar environmental conditions but differ in eutrophication levels. Such comparisons would provide valuable insights into the relative influence of nutrient enrichment on invasion dynamics and inform more targeted management strategies. However, our SWOT analysis is based on literature, expert knowledge, and interpretation rather than directly tested hypotheses. It also reflects the biological and ecological improbability of a proliferation rate equivalent to that observed in Lake Tana in the absence of eutrophication. Some of the points raised in the SWOT analysis would benefit from further experimental investigation to strengthen their evidential basis.

### Policy Implications

The findings of this study have important implications for freshwater management policies, both in Ethiopia and globally. First, interventions that rely solely on mechanical, chemical, or biological removal are unlikely to yield long-term success without addressing underlying nutrient enrichment. Policy frameworks should therefore integrate catchment-based nutrient management as a central component of invasive species control. This may include regulations on agricultural runoff, wastewater treatment, and land-use practices within lake catchments.

Second, the potential of water hyacinth biomass as a resource for circular bioeconomy initiatives such as compost, biogas, and industrial materials should be recognized in policy design. Policies that incentivize biomass utilization can reduce management costs while providing economic benefits to local communities.

Third, the study highlights the need for adaptive, evidence-based management policies that utilize remote sensing and other monitoring tools to evaluate the effectiveness of interventions over time. This approach can be scaled to other nutrient-enriched tropical freshwater systems, allowing policymakers to prioritize actions based on real-time ecological feedback.

Finally, integrating ecological insights with socio-economic considerations underscores the need for interdisciplinary policy development, involving environmental agencies, local communities, and researchers. This ensures that management measures are both ecologically effective and socially acceptable, providing a roadmap for long-term, sustainable control of aquatic invasive species.

Additionally, understanding the introduction pathways of water hyacinth into these freshwater systems remains a critical knowledge gap. Future research should focus on tracing these invasion routes, as such insights are essential for preventing new introductions and informing targeted, proactive management strategies.

## Conclusion

Our analysis from Lake Tana demonstrates that while large-scale removal of water hyacinth can achieve short-term success, it is unlikely to represent a sustainable long-term solution on its own. Despite removing over 75% of the plants, rapid regrowth and dispersal resulted in substantial reinvasion within one year. Our results suggest that single-control approaches (physical, chemical, or biological) re insufficient to fully address the persistence of water hyacinth, as its proliferation is closely linked to broader ecological degradation, particularly nutrient enrichment from the catchment runoff.

Using Lake Tana as a model system, our study provides insights that are relevant beyond the regional context and contribute to international scholarship on invasive aquatic plants in nutrient-enriched tropical freshwater systems. A sustainable solution must therefore combine continuous removal with improved catchment and waste management to reduce nutrient inputs. Moreover, integrating water hyacinth into circular bioeconomy initiatives, such as composting, biogas, and industrial applications, offers both ecological and socio-economic benefits, although the feasibility and scalability of such approaches require further empirical validation in different socio-ecological settings. Methodologically, our approach combining satellite monitoring, SWOT analysis, and socioecological system mapping offers a potentially replicable framework, though it should be interpreted as an exploratory and integrative assessment tool rather than a fully validated predictive model. This framework can support future studies assessing invasive aquatic plants and informing management strategies worldwide. Ultimately, addressing the root causes of eutrophication is crucial to restoring the health of freshwater ecosystems and ensuring long-term resilience against invasive aquatic weeds, such as water hyacinth. Overall, this study highlights water hyacinth as both an indicator of ecosystem nutrient status and a target for integrated, globally applicable management strategies. However, it is important to note that the SWOT analysis is based on literature, expert knowledge, and interpretation rather than directly tested hypotheses. Some of the SWOT-derived propositions remain conceptual and would benefit from targeted field experiments or longitudinal studies to strengthen their empirical basis.

Ultimately, addressing the root causes of eutrophication is crucial to restoring the health of freshwater ecosystems and ensuring long-term resilience against invasive aquatic weeds, such as water hyacinth. Overall, this study highlights water hyacinth as both an indicator of ecosystem nutrient status and a target for integrated, globally applicable management strategies, while emphasizing the need for context-specific validation before generalizing management recommendations globally.

## Supplementary information

Supplementary information

## Data Availability

We utilized satellite imagery.
