# Supplementary material for: Beyond Removal: Strategies for Sustainable Control of Water Hyacinth in Tropical Freshwater Ecosystems
Source: Environ Manage. 2026 May 20;76(6):187. doi: 10.1007/s00267-026-02494-1 (PMC13190500; doi:10.1007/s00267-026-02494-1)
Supplement: Supplementary file 1 — Supplementary information [file 267_2026_2494_MOESM1_ESM.pdf]

S1: Supplementary information

A system map for causes of water hyacinth proliferation

Figure 6 of the manuscript shows a system map of causal links and feedback loops among factors that impact freshwater bodies. The causal link maps are often referred as causal loop diagrams (CLDs). CLD is one of the tools used in the System Dynamics methodology to draw direction of causality among factors; factors that we identified as problem of interest. System Dynamics is a methodology based on modeling, simulation, and analysis that facilitates learning about systems that are complex and dynamic (i.e change with time). Also, it helps to understand the sources of policy resistance in such systems and in the redesign of more effective policies.

CLDs are used either to elicit knowledge that serve as base for detailed mathematical models or to summarize and communicate findings. In this paper, we used the CLDs with the later purpose. We used the CLDs to summarize and communicate our findings from the SWOT analysis and from the review of various literature that we conducted as part of this perspective paper.

Our SWOT analysis and the review of the literature clearly indicate that water hyacinth invasion of freshwaters represents a response to eutrophication rather than a process of invasion. The CLD in Figure 6 depicts this fact.

An increase in population puts pressure both in demand for more agricultural land and for developed land such as residential areas and associated infrastructure for transport, health, schooling to name a few. An increase in agricultural land would increase the food production provided than there would be sufficient capital investment that would facilitate the food production process. However, the competition for land from development activities and a degradation in the quality of soil nutrient due to intensive food production would reduce the amount of agricultural land that would be available for production next time round.

If there in an increase in food production, there would be more food that can be consumed. However, this accelerates the utilization of natural nutrients. The more the natural nutrients are consumed, the more the food product would demand for artificial nutrient to produce food. The more we apply artificial nutrients, the more would be the artificial nutrient runoff to the freshwater bodies unless there are efficient catchment management practice in place. In the

same token the more food we produce, the more it would be consumed. The more we consume the more would be the wase production, which would later end up in the freshwaters unless again there are good catchment and wase management practices.

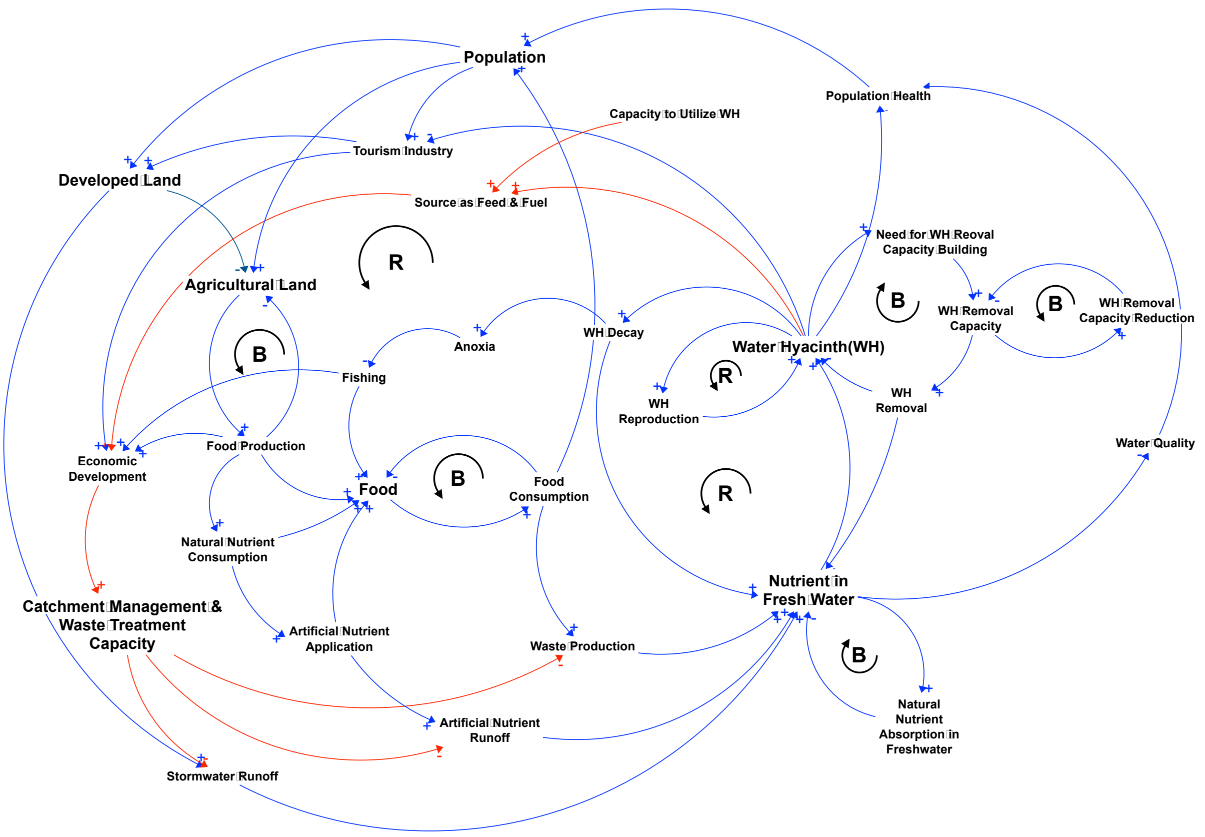

Figure 6. A system map for causes of water hyacinth proliferation in freshwater and potential policy for its reduction.
Selected feedback loops are labeled, with “R” indicating a reinforcing feedback loop and “B” indicating a balancing (a counteracting) feedback. The ‘+’and ‘-’ signs at the end of the arrows indicate the direction of increase or decrease in effect, respectively, given that everything else kept constant. Bolded variable names indicate key factors in causal loop diagram, where one can start to study the causal map. The red coloured links indicate potential policy options that can be considered.

Although an increase in food consumption has impact on the food available for next time round, it helps the population to grow more and demand again for more agricultural land and developed land. In the process, creating a positively reinforcing feedback loop that keeps the components under consideration to contentiously grow unless there is an external limiting factor such as the availability natural land or other developmental management policies.

An increase in developed land also increases stormwater runoff that adds nutrients to freshwater bodies. Only a certain amount of nutrient accumulated in the freshwater can be absorbed naturally, the rest would create a conducive environment for invasive species such as water hyacinth to flourish and hinder the natural nutrient absorption capacity in the freshwater.

This would increase the available nutrient in freshwater next time around. The water hyacinth partly helped by their short life cycle and their ability to produce thousands of seeds, and partly by their asexual propagation can easily and quickly proliferate and invade freshwater bodies. Furthermore, the decay of water hyacinth do not only added nutrient into the freshwater to help the water hyacinth flourish next time around, but also create anoxia condition that kills fish and other aquatic animals, creating problem on food available for consumption and economic issues on the fishing industry.

Water hyacinth do not only have negative impacts. If there is a proper policy in place and if the water hyacinth are at manageable level, they could sever as source of feed for animals and as fuel too. Also, they could be used for making compost, paper, as well as furniture. However, if they proliferate at very high level, they will have significant impact on the tourism industry as well, blocking navigations. If unchecked, water hyacinth in freshwater also serve as breading places for vectors such as mosquitos that might have significant impact on the population health. Similarly, freshwater bodes invaded with water hyacinth often have poor water quality that intern have impact on the population health.

An increase in water hyacinth in freshwater bodies would require significant capacity for removal. The utilization of the capacity would reduce the available removal capacity next time round unless there is a way to replenish the removal capacity externally. The removal of the water hyacinth can reduce their reproduction and propagation capacity next time round, which would reduce the nutrient level added into the fresh water next time round there by improving the quality of the freshwater. However, this is only possible if the water hyacinth removal is coupled with effective catchment and waste management practices. Otherwise, as can be seen in the CLD (Figure 6), removing water hyacinth alone would not reduce the influx of nutrient into the freshwater. As long as there is influx of nutrient from the catchment area into the freshwater bodies, invasive species such as water hyacinth will keep flourishing. If there are good catchment and waste management practices, however, which can be coupled with water hyacinth removals, the capacity that would be required to remove the water hyacinth from the freshwater next time round would reduce, there by reducing the need for the external intervention and increasing the quality of freshwater bodies.
